# Incidental Findings Following Dental Implant Procedures in the Mandible: A New Post-Processing CBCT Software Analysis

**DOI:** 10.3390/diagnostics14171908

**Published:** 2024-08-29

**Authors:** Marcel da Silva Garrote, Ana Helena Gonçalves de Alencar, Cyntia Rodrigues de Araújo Estrela, Lucas Rodrigues de Araújo Estrela, Mike Reis Bueno, Orlando Aguirre Guedes, Carlos Estrela

**Affiliations:** 1 Department of Endodontics, School of Dentistry, Federal University of Goiás, Goiânia 74605-020, Brazil; ahga@ibest.com.br (A.H.G.d.A.); estrela3@terra.com.br (C.E.); 2 Department of Oral Biology, School of Dentistry, Evangelical University of Goiás, Anápolis 75083-515, Brazil; estrelacyntia@gmail.com (C.R.d.A.E.); orlandoaguedes@gmail.com (O.A.G.); 3 Department of Preventive and Restorative Dentistry, School of Dentistry, São Paulo State University, Araçatuba 16015-050, Brazil; estrelalucas4@gmail.com; 4 Center for Radiology and Orofacial Images, Diagnostic Imaging Center, Cuiabá 78043-272, Brazil; mikebueno@terra.com.br

**Keywords:** cone–beam computed tomography, diagnosis, dental implant, incidental findings

## Abstract

**Background/Objectives**: The aim of this study was to evaluate incidental findings in the mandible after the placement of dental implants using a new cone–beam computed tomography (CBCT) software. **Methods**: The initial sample consisted of 2872 CBCT scans of patients of both sexes. The parameters evaluated in this study were the location of the implants in the mandible, implant length, anatomical relationship of the implant with the mandibular canal, presence or absence of damage to the adjacent teeth, presence or absence of implant fractures, and presence or absence of bone support. Fisher’s exact test was performed to compare the variables. The significance level was set at *p* = 0.05. **Results**: Out of 2872 CBCT scans, 214 images of patients with an average age of 44.5 years were included. The most frequent location of the implants was the posterior region (93.5%), with 54% of the implants having a length between 9 and 14 mm. It was found that 92% of the implants were positioned above the mandibular canal. Damage to adjacent teeth was observed, with no correlation with the implant positioning (*p* = 1.000). In 100% of cases of implants in the anterior region, there was bone support. Fracture was observed in 1.7% of implants with a length between 9 and 14 mm. **Conclusions**: The installation of implants in the mandible occurs more frequently in the posterior region, with a high presence of bone support and a low incidence of damage to adjacent teeth, anatomical structures, and fractures.

## 1. Introduction

Rehabilitation with dental implants presents high success rates [1,2,3]. However, accidents and complications are not uncommon, often resulting from inappropriate indications and insufficient planning [4,5,6,7,8,9].

The most common surgical technique error is related to implant malpositioning and angulation, compromising both aesthetics and biomechanics [10,11]. Additionally, cases involving inadequate spacing between the implant and adjacent structures, cortical bone perforations, and penetration into anatomical structures can also contribute to treatment failure with implants [12].

The posterior region of the mandible, due to the presence of the inferior alveolar nerve and the submandibular fossa, represents a high-risk zone during implant installation, due to the potential for injury to the neurovascular bundle and perforation of the lingual cortex [13]. Inadvertent manipulation of the lingual cortex can cause arterial damage, leading to subsequent hematoma formation in the submandibular and sublingual spaces [14,15].

The consequences of invading the mandibular canal can range from paresthesia to hyperesthesia. Persistent neurosensory disturbances are rare and, in most cases, reversible; however, the outcomes can be unpredictable [10]. The exact prevalence of sensory disturbances is not known, but some studies report sensory changes in the lips following dental implants at an average rate of 8 to 24% [4].

In the anterior region, inadequate spacing between the implant and the adjacent tooth or implant is more common [12]. Perforations in adjacent teeth can cause endodontic injuries and lead to the development of chronic pathologies [10,16]. Injuries to the incisive canal or accessory neurovascular canals can result in neurosensory disturbances and pain [13,17,18].

Perforations of the buccal cortex can lead to dehiscence and fenestration, compromising aesthetics and increasing the risk of peri-implantitis [16,17]. Similarly, perforations of the lingual cortex in the mandible, including the submandibular fossa, have the potential to become life-threatening due to sublingual hematoma that may obstruct the airway [10,13,17].

To avoid accidents and complications, proper pre-surgical planning is essential [17]. Clinical evaluation and cone–beam computed tomography (CBCT) images are recommended as a dynamic guide for planning the implant position [4,9,10,14,19,20]. These images allow for more precise and three-dimensional (3D) measurements of the bone site to be implanted, optimizing the identification of anatomical structures and their variations, as well as providing information on bone morphology [1,12,17,21,22]. CBCT provides images obtained in a single rotation, showing minimal metal artifacts, with reduced cost, ease of handling, and low radiation doses compared to multi-slice CT protocols [9,18,19,23].

CBCT presents notable limitations in the postoperative evaluation of dental implants due to the significant production of metal artifacts [9]. These artifacts, generated by the presence of metallic components, severely compromise the image contrast and visual quality, thereby diminishing the accuracy of the analysis in CBCT scans [9,10,12,19,22]. The distorted imaging resulting from these artifacts can lead to critical errors in the interpretation of the images, adversely affecting clinical and diagnostic decision-making processes [19,22,24]. Despite numerous attempts to mitigate or eliminate these metal artifacts, particularly in cone–beam computed tomography (CBCT), complete eradication remains unachieved [19,24].

The use of state-of-the-art technologies for analyzing the implant treatment site through dynamic imaging is essential to increase procedural accuracy and predictability of success. An example is e-Vol DX, new free-access software associated with a DICOM viewer. This software includes advanced features such as BAR (Blooming Artifact Reduction) filters, which enhance image sharpness and resolution, providing crucial information to improve decision-making from the initial stage of treatment planning [19,24].

The location of the mandibular canal is crucial for establishing boundaries during surgical planning. However, even with tomography, its visualization is difficult when the canal is not corticalized [4,11,25,26]. Nevertheless, it is easier to visualize the canal in more posterior regions with the aid of this examination, enabling its identification by tracing its path through differences in density [14].

Given the risks of failure in implant treatments and the technological advancements and new techniques frequently indicated for dental approaches, prior evaluation of the mandibular region where dental implants are intended to be placed becomes essential for planning [9]. Intraoral imaging exams are essential tools for guiding treatment plans in modern dentistry [27,28,29]. To the authors’ knowledge, no study has been conducted to assess the incidental findings following dental implant procedures using CBCT and e-Vol DX software [23,24]. Thus, the present study aimed to investigate the incidental findings following dental implant procedures in the mandible using this new post-processing CBCT software.

## 2. Materials and Methods

This study was reviewed and approved by the Research Ethics Committee on Human Beings of the Evangelical University of Goiás (CAAE 71055623.0.00005076).

For this research, CBCT scans from the database of the Center for Radiology and Orofacial Imaging (CROIF), a private clinic located in Cuiabá, Brazil, were selected. These scans were initially conducted for diagnostic purposes from January 2015 to December 2020. A convenience sample of 2872 CBCT scans from patients of both sexes, aged between 18 and 80 years old, was initially selected.

The inclusion criteria for selecting CBCT scans in this study were based on choosing images that showed dental implants located in the mandibular region, along with the adjacent teeth to the implants. Images displaying bone alterations related to benign or malignant diseases or neoplasms in the mandible were excluded from the study.

The tomographic images in this study were acquired using a PreXion 3D scanner (Prexion 3d Inc., San Mateo, CA, USA), following a standard protocol with the following specifications: slice thickness of 0.100 mm, dimensions of 1170 mm × 1570 mm × 1925 mm, field of view (FOV) of 56.00 mm; voxel size of 0.108 mm, exposure time of 37 s (16 bits), tube voltage of 90 kVp, and tube current of 4 mA. The images were analyzed using e-Vol DX software (CDT Software; São José dos Campos, SP, Brazil), running on a workstation with an Intel i7-7700K processor clocked at 4.20 GHz (Intel Corp., Santa Clara, CA, USA), NVIDIA GeForce GTX 1070 graphics card (NVIDIA Corporation, Santa Clara, CA, USA), Dell P2719H monitor with a resolution of 1920 × 1080 pixels (Dell Technologies Inc., Round Rock, TX, USA), and Windows 10 Pro operating system (Microsoft Corp., Redmond, WA, USA). The use of high-resolution images ensured the necessary diagnostic accuracy for the study.

All tomographic scans were standardized to align the implants axially. Additionally, the sagittal and coronal planes were used to position the long axis of the sample transversely to the ground, correcting for any parallax error.

The parameters evaluated in this study were defined based on Safi et al. [17] and included the following:

1. Positioning of dental implants: 0: implant located in the anterior right region of the mandible (right central incisor to right canine); 1: implant located in the anterior left region of the mandible (left central incisor to left canine); 2: implant located in the posterior right region of the mandible (right first premolar to right second molar); and 3: implant located in the posterior left region of the mandible (left first premolar to left second molar).
2. Length of dental implants: 0: implant length less than 9 mm; 1: implant length between 9 mm and 14 mm; and 2: implant length greater than 14 mm.
3. Anatomical relationship between the implant and mandibular canal: 0: implant above the upper limit of the mandibular canal by 1–2 mm; 1: implant in contact with the mandibular canal; and 2: implant within the mandibular canal (1 mm or more)
4. Damage to adjacent teeth to the implants: 0: no damage to adjacent teeth; 1: damage to the tooth located anteriorly to the implant; and 2: damage to the tooth located posteriorly to the implant.
5. Implant fracture: 0: no fracture in the implant; and 1: presence of fracture in the implant
6. Bone support for the implant: 0: absence of bone support for the implant; and 1: presence of bone support for the implant.

The analyses were conducted sequentially by two examiners with over a decade of experience in procedures associated with CBCT scans. Before commencing the analyses, the examiners underwent calibration by reviewing scans based on the same inclusion and exclusion criteria used in the study. In case of disagreement, a third equally qualified examiner was consulted to make the final decision.

The statistical analysis was performed using IBM SPSS for Windows 20.0 software (IBM Corp., Armonk, NY, USA). Qualitative variables were described using frequencies and percentages, along with the presentation of the 95% confidence interval for the proportion in each category. Fisher’s exact test was used to compare the variables. A significance level of 5% was adopted for all comparisons. Inter-examiner agreement was evaluated using the kappa coefficient in 10% of the sample.

## 3. Results

**Figure 1:**
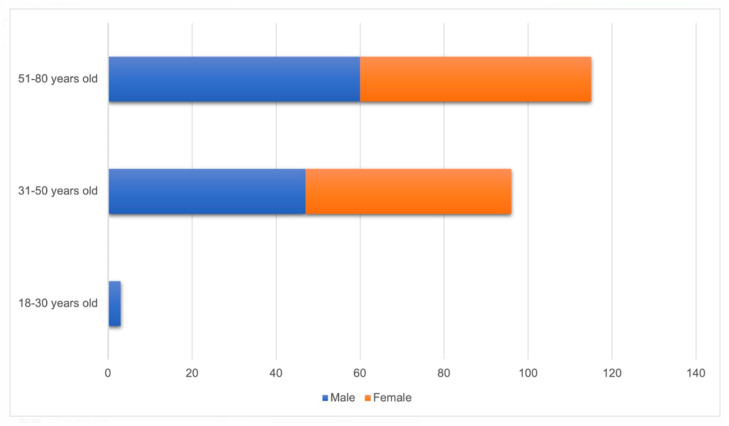
Distribution of patients according to sex and age group (n = 214).

The kappa value was 0.87, which indicated an excellent degree of inter-examiner agreement. A total of 214 CBCT images were included in this study. Most patients were male (51.4%), and the most frequent age range was from 51 to 80 years (53.8%). Detailed characteristics of the sample and incidental findings are presented in Table 1 and Figure 1.

**Figure 2:**
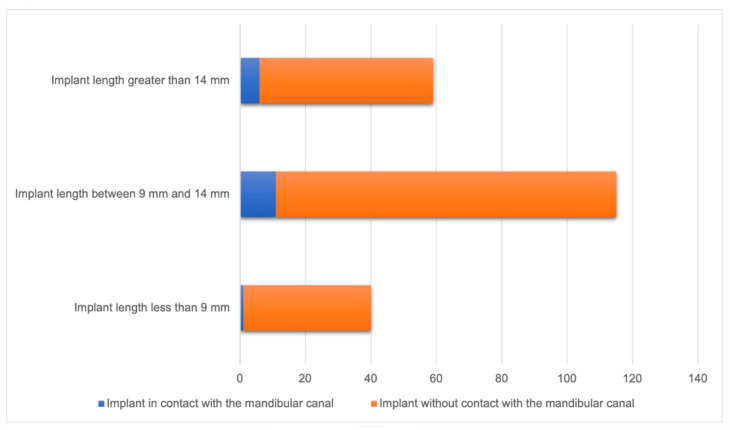
Relationship between implant length and mandibular canal (n = 214).

Among implants with a length of less than 9 mm, one (2.5%) had contact with the mandibular canal. For implants with a length between 9 mm and 14 mm, 11 (9.6%) showed contact with the mandibular canal, while for implants longer than 14 mm, 6 (10.2%) also had contact with the mandibular canal. There was no observed association between implant length and invasion of the mandibular canal (*p* = 0.357) (Figure 2).

**Figure 3:**
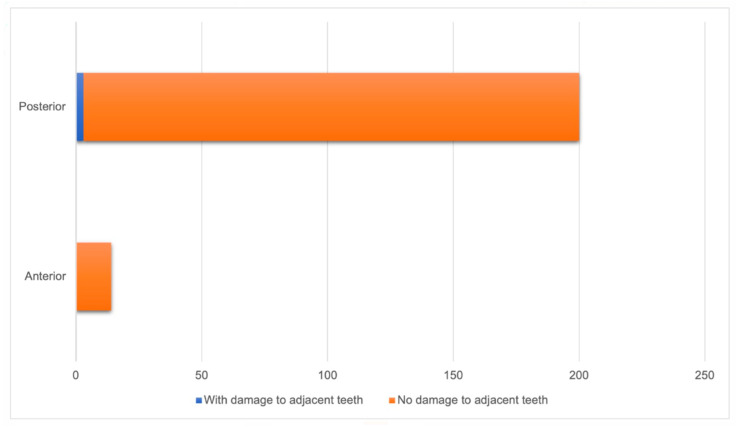
Relationship between implant positioning (anterior or posterior region) and occurrence of damage to adjacent teeth (n = 214).

None of the implants installed in the anterior region caused damage to adjacent teeth, while three (1.4%) of the implants in the posterior region resulted in damage to adjacent teeth. No statistically significant difference was found between teeth with damage and the implant position (*p* = 1.000) (Figure 3).

**Figure 4:**
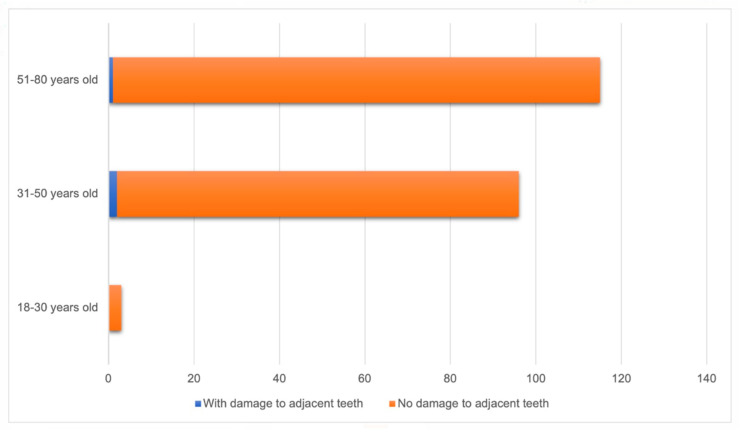
Relationship between age group and occurrence of damage to adjacent teeth (n = 214).

None of the implants performed on patients aged 18 to 30 years caused damage to adjacent teeth. Among implants performed on patients aged 31 to 50 years, two (2.1%) resulted in damage, while among those performed on patients aged 51 to 80 years, one (0.9%) caused damage to adjacent teeth. No statistically significant difference was found between the groups (*p* = 0.609) (Figure 4).

**Figure 5:**
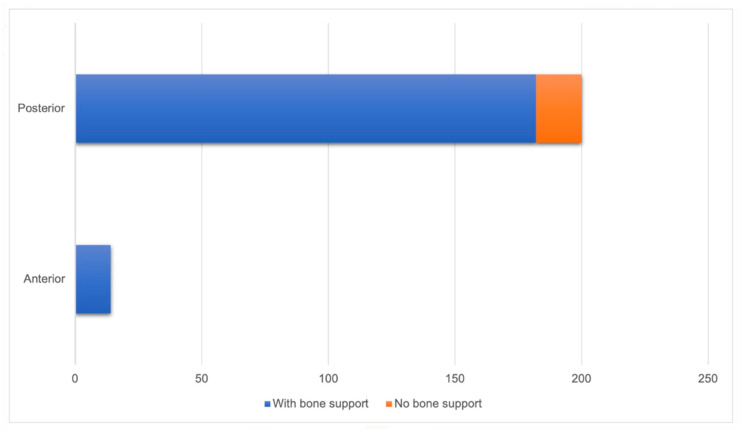
Relationship between implant positioning and bone support (n = 214).

All anterior implants (100%) had bone support, while among posterior implants, 91% had bone support, with no statistically significant difference between them (*p* = 0.614) (Figure 5).

**Figure 6:**
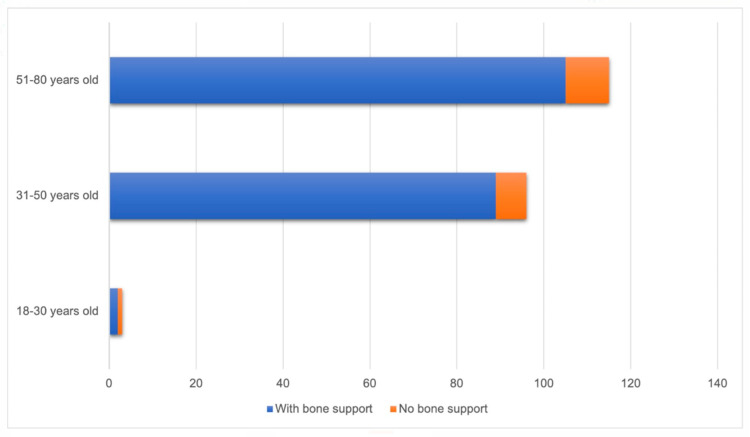
Relationship between age and bone support (n = 214).

There was no statistically significant difference in bone support among age groups (*p* = 0.254). Among patients aged 18 to 30 years, 66.7% had bone support for the implant; in the age group of 31 to 50 years, 92.7% exhibited bone support; and among patients aged 51 to 80 years, 91.3% (Figure 6).

**Figure 7:**
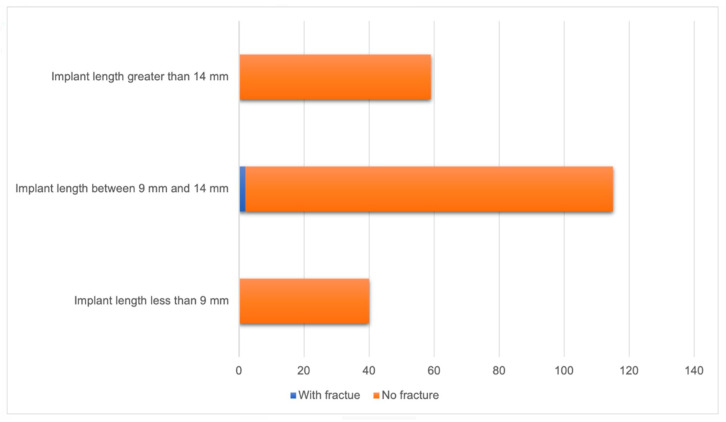
Relationship between implant length and implants fracture (n = 214).

No fractures were observed in implants with lengths less than 9 mm or greater than 14 mm. However, among implants with lengths between 9 mm and 14 mm, two (1.7%) fractures were found. There was no statistically significant relationship between implant length and occurrence of fractures (*p* = 0.702) (Figure 7).

## 4. Discussion

Of the 214 exams evaluated, most patients were male, and the most frequent age range was from 51 to 80 years. The most frequent location of implants was in the posterior region of the mandible, with most implants positioned 1 to 2 mm away from the mandibular canal. It was observed that the most common implant length was between 9 and 14 mm (53.7%), aligning with the findings of Safi et al. [17], where 71% of the sample fell within this length range. Damage to adjacent teeth was observed in 1.4% of cases, while 0.9% showed implant fractures and 8.4% exhibited a lack of bone support for the implant.

Complications following implant installation are expected in approximately 14% of cases. Preoperative clinical evaluation and radiographic assessment of bone quality and quantity are crucial for treatment success [10,12,17,20].

CBCT (cone–beam computed tomography) is the modality of choice for implant planning as it allows precise definition of shape, morphology, and bone quantity [20]. This technology assists in precise planning to avoid angulation errors in implants [9,10,12,14,17]. High-quality images should capture all radiographic information accurately, without artifacts that could compromise interpretation [19].

Implants in contact with the mandibular canal can result in trauma to the neurovascular bundle, causing various degrees of sensory alterations [18]. These injuries may occur due to the use of long implants, inappropriate angulation, or anatomical variations [30]. Additionally, displacement of implants in the posterior region of the mandible due to low bone density can lead to nerve injuries and paresthesia [10,17,30].

Recent studies have highlighted the importance of using surgical guides in implantology to reduce complications and enhance procedural accuracy [31,32]. Flügge et al. [32] demonstrated that 3D-printed surgical guides significantly improve the precision of implant placement, which can minimize the risk of damaging adjacent anatomical structures, such as the mandibular canal.

The absence of bone support for the implant is a relatively common complication, often resulting from implants with inadequate angulation. This condition promotes peri-implant bone loss, which can affect aesthetic outcomes and facilitate bacterial colonization, potentially leading to implant failure [10,12]. Implants that perforate adjacent structures have a higher prevalence of exposed threads. Gaeta-Araujo et al. [10] observed that exposed threads and implant failure are more common in the posterior region. This phenomenon is more frequent in the posterior region due to the high masticatory load in this area of the jaws and inadequate bone height.

Despite a low incidence of damage to adjacent teeth observed in the present study, Ribas et al. [12] noted that this is a primary complication, with a rate of 18.8% among the cases analyzed in their study. This can be attributed to their specific focus on cases where there was contact with adjacent teeth, resulting in injury. Ribas et al. [12] also discussed the importance of adequate distance between teeth and implants, which should be at least 1.5 mm, as established in the literature. Failure to respect this distance can lead to future problems due to inadequate nutrition of the bone crest.

Implant fracture is a significant mechanical complication that can occur due to metal fatigue or excessive occlusal force [17]. The causes and mechanisms leading to implant failure depend on multiple factors. These include implant location, diameter and height of the implant, bone quality and quantity, attention to vital structures, and operator techniques and skills [17]. Interestingly, the literature indicates that anatomical variations are not the primary drivers of high implant failure rates. Studies suggest that while failures are common, there is not a significant correlation with anatomical variations in the sample. This suggests that mechanical and technical factors play a more predominant role, as also observed in the present study.

The present study did not identify a statistically significant relationship between implant length and invasion of the mandibular canal (*p* > 0.05), consistent with the findings of Safi et al. [17]. The relationship between implant dimensions and success rates remains controversial in the literature. Although a higher number of failures is often associated with short and narrow implants, it cannot be asserted that these dimensions are the primary factors responsible for low implant survival rates [10].

As observed by Safi et al. [17], this study did not find a statistically significant relationship between age, sex, and implant malpositioning. In the present sample, only one case presented with a lingual plate fracture, located in the posterior region of the left mandible. This area is particularly vulnerable due to the presence of the submandibular fossa, an anatomical depression of the mandible accommodating the submandibular gland, as well as branches of the sublingual and submental arteries. Injuries to these structures can result in submandibular hematoma formation, tongue elevation, and possible obstruction of the upper airways [14]. De Souza et al. [14] demonstrated that the depth of the submandibular fossa can range from 0.0 mm (no concavity) to 5.0 mm (maximum concavity), being more pronounced in the posterior regions of the mandible. Parnia et al. [33] indicate that when the depth of the fossa exceeds 2.0 mm, the risk of lingual plate perforation during implant installation can reach up to 80%.

Computed tomography (CT) had significant limitations in the postoperative evaluation of dental implants. The main reason was the substantial production of artifacts due to the presence of metal [9,10,12,19,22]. These metal artifacts compromise image contrast, altering visual quality and reducing the accuracy of analysis in CT scans [19,24]. The presence of these artifacts can lead to critical errors in image interpretation, directly impacting clinical and diagnostic decision-making [19,22,24].

e-Vol DX software provides a significant advantage by offering dynamic navigation through computed tomography (CT) images, minimizing artifact production. This feature is crucial for precise pre-surgical planning, essential for preventing complications associated with improper positioning of dental implants [19]. Inadequate planning can be influenced by both the professional’s experience and the imaging modality used [10,19]. In this context, guided surgery has emerged as an effective solution, reducing surgical complications that may arise from operational errors [10,17].

e-Vol DX software offers another advantage with its compatibility across all CT scanners and its ability to export files in DICOM (Digital Imaging and Communications in Medicine) format. This format ensures superior quality in brightness, contrast, sharpness, thickness, and noise reduction parameters. Additionally, the software incorporates advanced filters that prevent image quality loss, enhancing saturation and brightness in specific areas while reducing illumination in areas without image data. These concepts, adapted from digital photography, are crucial for artifact reduction [19,24]. Furthermore, e-Vol DX provides high-quality images with protocols that feature short exposure times, thereby resulting in reduced radiation doses [19].

The precise localization of the mandibular canal is crucial for surgical planning in dental implant procedures, as it defines the apical limit of the intervention in the mandible. Identifying the canal can be particularly challenging in cases where the structure is poorly corticated, which is common in patients with poorly mineralized trabecular bone. In such situations, the absence of clear corticalization of the mandibular canal makes its visualization difficult [14]. The study by De Souza et al. [14]. highlighted that identifying the mandibular canal becomes even more complex as it approaches the mental foramen. This difficulty is exacerbated in elderly patients due to changes in bone density and mineralization. In these cases, the e-Vol DX software proves especially useful. Its filters and advanced features enable the organization and detailed analysis of information and images, facilitating the identification of patterns and common deviations in areas where corticalization is deficient. This capability is particularly beneficial for identifying the mandibular canal in patients with low corticalization [19].

The present study has some important limitations. The absence of bone support around the implant can occur at different times: during installation, due to inadequate planning, when the surgeon identifies insufficient bone thickness at the time of surgery and decides to leave the implant without support on all walls; or post-installation, as a result of a physiological process of alveolar bone resorption. In our study, it was not possible to determine the interval between implant installation and cone–beam computed tomography (CBCT) examination, which could influence the assessment of osseointegration. Another significant limitation was the lack of detailed clinical information regarding symptoms or complaints associated with CBCT findings, restricting the correlation between imaging findings and clinical manifestations.

Certainly, e-Vol DX software represents an invaluable tool for enhancing the quality of cone–beam computed tomography (CBCT) scans by facilitating diagnoses through the reduction of common metal artifacts inherent in such examinations. Originally developed to improve diagnostics in endodontics, this software meets the need for enhanced diagnostic accuracy across various fields, including implant dentistry, which often encounters similar challenges with metal artifacts. This study aimed to extrapolate the advantages and applications of e-Vol DX to other areas, exploring how it can mitigate metal artifact interference and improve image interpretation. Further studies are needed to comprehensively investigate the benefits of this software, correlating imaging findings with specific clinical data.

## 5. Conclusions

Implant placement in the mandible occurred most frequently in the posterior region. Adequate bone support was observed with a low incidence of complications.

## Figures and Tables

**Table 1:** Incidental findings following dental implant procedures in the mandible (n = 214).

Characteristics	n (%)	95% IC
Sex		
Female	104 (48.6)	41.7–55.5
Male	110 (51.4)	44.5–58.3
Age		
18 to 30 years old	3 (1.4)	0.3–4.0
31 to 50 years old	96 (44.8)	38.1–51.8
51 to 80 years old	115 (53.8)	46.8–60.6
Implant position		
Implant located in the anterior region of the right mandible	9 (4.2)	1.9–7.8
Implant located in the anterior region of the left mandible	5 (2.3)	0.8–5.4
Implant located in the posterior region of the right mandible	98 (45.8)	39.0–52.7
Implant located in the posterior region of the left mandible	102 (47.7)	40.8–54.6
Implant length		
Implant length less than 9 mm	40 (18.7)	13.7–24.6
Implant length between 9 mm and 14 mm	115 (53.7)	46.8–60.6
Implant length greater than 14 mm	59 (27.6)	21.7–34.1
Anatomical relationship between implant and mandibular canal		
Implant short of the mandibular canal (1 to 2 mm)	196 (91.6)	87.0–94.9
Implant in contact with the mandibular canal	18 (8.4)	5.1–13.0
Implant within the mandibular canal (1 mm or more)	0 (0)	0–0
Occurrence of damage to adjacent teeth		
No damage to adjacent teeth	211 (98.6)	96.0–99.7
Damage to the tooth located anterior to the implant	0 (0)	0.1–3.3
Damage to the tooth located posterior to the implant	3 (1.4)	0.01–2.6
Occurrence of implant fracture		
No fracture in the implant	212 (99.1)	96.7–99.9
Fracture in the implant	2 (0.9)	0.1–3.3
Bone support for the implant		
Absence of bone support for the implant *	18 (8.4)	5.1–13.0
Presence of bone support for the implant	196 (91.6)	87.0–94.9

* 1 case of lingual bone plate fracture. 95% CI: 95% confidence interval.

## Data Availability

The data are available in this article.
